# Improving the Accuracy of Direct Geo-referencing of Smartphone-Based Mobile Mapping Systems Using Relative Orientation and Scene Geometric Constraints

**DOI:** 10.3390/s17102237

**Published:** 2017-09-30

**Authors:** Naif M. Alsubaie, Ahmed A. Youssef, Naser El-Sheimy

**Affiliations:** Geomatics Engineering Department, University of Calgary, Calgary, AB T2N 1N4, Canada; ahmed.youssef1@ucalgary.ca (A.A.Y.); elsheimy@ucalgary.ca (N.E.-S.)

**Keywords:** mobile mapping system, close range photogrammetry, smartphone, low-cost navigation systems, relative orientation, geometric constraints

## Abstract

This paper introduces a new method which facilitate the use of smartphones as a handheld low-cost mobile mapping system (MMS). Smartphones are becoming more sophisticated and smarter and are quickly closing the gap between computers and portable tablet devices. The current generation of smartphones are equipped with low-cost GPS receivers, high-resolution digital cameras, and micro-electro mechanical systems (MEMS)-based navigation sensors (e.g., accelerometers, gyroscopes, magnetic compasses, and barometers). These sensors are in fact the essential components for a MMS. However, smartphone navigation sensors suffer from the poor accuracy of global navigation satellite System (GNSS), accumulated drift, and high signal noise. These issues affect the accuracy of the initial Exterior Orientation Parameters (EOPs) that are inputted into the bundle adjustment algorithm, which then produces inaccurate 3D mapping solutions. This paper proposes new methodologies for increasing the accuracy of direct geo-referencing of smartphones using relative orientation and smartphone motion sensor measurements as well as integrating geometric scene constraints into free network bundle adjustment. The new methodologies incorporate fusing the relative orientations of the captured images and their corresponding motion sensor measurements to improve the initial EOPs. Then, the geometric features (e.g., horizontal and vertical linear lines) visible in each image are extracted and used as constraints in the bundle adjustment procedure which correct the relative position and orientation of the 3D mapping solution.

## 1. Introduction

Over the past two decades, mobile mapping systems (MMS) have been a vital source of direct geo-referenced data, which can be used for a variety of applications (e.g., mapping, 3D modeling, highway inventory, engineering projects, and Geographic Information System (GIS) data updates). Although land-based MMS is one of the main sources for acquiring direct geo-referenced data, there are many drawbacks to using the current MMS (i.e., their large size and complexity, as well as its high cost due to the use of expensive Inertial Measurements Unit (IMU) and GNSS receivers) which have restricted their widespread adoption in the survey and mapping industries. Consequently, the market for land-based MMS is small, and the existing MMS typically are operated by the companies or institutions that built them, which unfortunately means that their more efficient data collection is not available for wider use [1]. The research trend now is toward a more cost-effective, less complex, and time-efficient MMS. The accuracy of direct geo-referenced data depends on the intended application; for example, inventory applications require one to two-meter accuracy. This paper specifically focuses on the development of a low-cost MMS based on smartphone technology. 

The proposed system overcomes the drawbacks of the current MMS (i.e., large size, complexity, and high cost) that have restricted their widespread adoption in disciplines which demand meter-level accuracies (e.g., documentation, inventory, surveying, and mapping). The development of such a system will satisfy the demand for a MMS that can compete both in cost and in user-friendliness with current terrestrial photogrammetry. The proposed system does not attempt to replace existing MMS, rather, it offers new low-cost alternative for applications requiring one to five meters accuracy. The GPS receivers used in most of the current smartphones (e.g., iPhone, Samsung, and HTC) have poor positioning accuracy. Furthermore, the MEMS sensors, especially gyroscopes, will accumulate position drift over short time because of their high signal-to-noise ratio over time. Furthermore, magnetometers can be easily disturbed by the presence of metallic objects in its vicinity. Although these sensors offer the ability to acquire direct geo-referencing data, their low-level sensor measurements can lead to inaccurate exterior orientation parameters (EOPs) which, in turn, decrease the mapping accuracy of the system. These erroneous EOPs must be corrected before calculating the final 3-D mapping coordinates of the points of interest. Therefore, a relative orientation approach is introduced in this paper to refine the initial EOPs. Then geometric features (e.g., straight vertical and horizontal linear features) are extracted, matched, and used to impose constraints on the object space calculation and adjustment inside the bundle adjustment model. The coplanarity constraint [2] is a well-known method for relative orientation estimation through an iterative process. However, the coplanarity constraint using least square adjustment requires good quality approximation of the unknown relative EOPs due to the highly iterative process based on the nonlinear nature of the model [3]. To solve this problem, several past studies introduced closed-form solutions to overcome these issues, such as eight-point [4,5] and five-point [6] algorithms. Similarly, the Structure from Motion (SfM) algorithm was originally developed by the computer vision community for solving 3D reconstruction problem using these closed-form solutions. SfM is commonly being used now in photogrammetry for automatic computation of initial relative EOPs [3,7,8].

## 2. Related Works

The process of integrating geometric constraints into bundle adjustment has generated a lot of interest within the photogrammetric community. McGlone [9,10] incorporated geometric constraints into bundle adjustment to improve the accuracy and precision of a detailed site model generated using multiple oblique airborne imagery. The author used the coplanarity condition that involved any number of object space points, which were used to fix the line or plane parameters and then were used to constrain the bundle adjustment. The effectiveness of this method was demonstrated in an experiment using airborne images of model-board buildings. Geometric constraints typically also are used is in the camera calibration process. Habib et al. [11] integrated geometric constraints in a bundle adjustment for self-calibration using straight lines and coplanarity conditions. The idea was based on the fact that, in the absence of camera distortions, the perspective projection of straight lines in object space must yield to a straight line in the image space. The idea behind this method is based on the coplanarity condition, more specifically, using stereo-pair imagery where three vectors satisfy the coplanar condition such that the first vector is connecting the perspective center to the first point along an object space straight line; the second vector is connecting the perspective center to the first point along an object space straight line; and the third vector is connecting the perspective center to any intermediate point along the image space line. Gerke [12] evaluated the use of geometric constraints to reduce the number of ground control points (GCPs) needed for indirect sensor orientation, whereby the geometric scenes (linear horizontal, vertical, and right-angle) that were visible in overlapping imagery were integrated into the bundle adjustment procedure along with some GCPs for the EOPs and Interior Orientation Parameters (IOPs) recovery. The author focused mainly on performing multi-camera self-calibrations by comparing the presence and the absence of certain distortion parameters for camera calibration in different scenarios as well as constraining the indirect orientation. This method was evaluated using two different airborne datasets, one acquired using a pictometry system and the other from a UAV equipped with a consumer digital camera. The author demonstrated the suitability of incorporating geometric constraints for reducing the need of well-distributed GCPs. Geometric constraints were also used to improve bad network geometry in past studies. For example, Zhang et al. [13] included planarity constraints and the constraints of highly correlated EOPs in a bundle adjustment to overcome the weak geometry connection of an image network to generate a precise ortho-image of the Dunhuang wall painting. However, this wall is a near-planar wall surface and the forward overlap between the network images were less than 60%, which produced a strong correlation between the EOPs and led to a singular normal matrix and increased the error propagation of the adjustment model. Therefore, the planarity constraints of the painting were used by the authors to control the error propagation by improving the geometric connection. The results of their experiment confirmed the effectiveness of these constraints for improving the stability and accuracy of the adjustment model. Likewise, geometric constraints were used to improve the overall accuracy of direct geo-referencing. El-Sheimy [14] used known geometric constraints, such as straight lines to place additional constraints on the calibration of a land-based MMS. All the studies above show the benefits of integrating geometric constraints for different photogrammetric applications; however, no studies to date have used these constraints to improve direct geo-reference using low-cost motion navigation sensors.

Current smartphones integrate on one platform low-cost GPS receivers, barometers, cameras, IMUs, and magnetometers, which are the ideal MMS components and have the key advantages of low cost, small size, and easy availability. A limited number of studies in the literature have investigated the use of smartphones for mapping applications. Al-Hamad and El-Sheimy [15,16,17] developed an innovative workflow for using smartphones as a low-cost MMS, whereby, the relative accuracy of the captured images’ EOPs was improved using a vision- based epipolar geometry technique. The epipolar line along with the automated matched points were used as a constraint to enhance the relative position and orientation of each captured image with respect to the first captured image. Although this work successfully illustrated that “Mobile Mapping Using Smartphones” is a potentially promising low-cost solution, the accuracy of the entire solution is governed by the accuracy of the first image’s EOPs, which are not usually accurate. Therefore, potential of a more robust method is needed. Alsubaie and El-Sheimy [18] introduced the potential of generating a direct geo-referenced image-based 3D point cloud using smartphones. This study demonstrated the suitability of incorporating geometric constraints to reduce the need for the conventional well distributed number of GCPs to improve direct geo-referencing using the initial EOPs directly from smartphones. However, the accuracy of the GPS chipset embedded in smartphones can exceed 10 m in multi-path conditions. However, only a few recent releases of the android smartphones (e.g., Nexus 9) allow access to the raw GPS measurements [19], while most of the Android and iOS smartphones do not provide raw measurements. Such limitation makes it impossible to apply differential GPS method without hardware modification [20]. 

**Figure 1:**
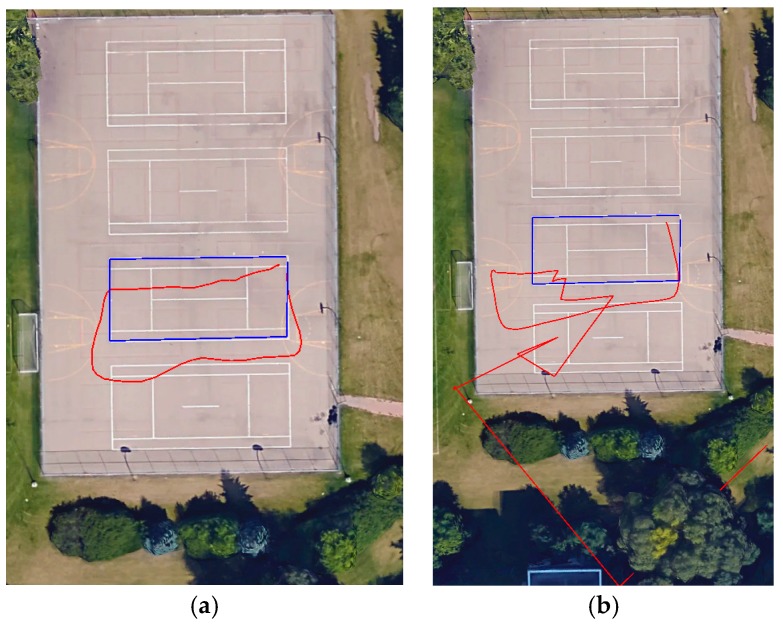
(**a**) First test; (**b**) Second test @ Google.

Figure 1 illustrates the accuracy of the GPS chipset embedded in the iPhone 6 for a known shape. The blue color trajectory represents the reference trajectory of a tennis court and the red color is the GPS solution. Two tests were collected over the same trajectory with a short time difference between them. As shown in Table 1, the total distance error in the second test was not acceptable for both navigation and mapping applications, while the total distance error in the first test is within the expected accuracy of the GPS single point positioning solution. These tests clearly show the challenge associated that can be faced when relying only on smartphone’s GPS chipset for mapping applications. Therefore, the objective of this paper was to use any smartphone as a MMS by overcoming the GPS limitation and the IMU drift issue associated with most smartphones.

Furthermore, the errors of the MEMS sensors, used in smartphones, typically change over time (due to changing temperature) and from turn-on to turn-on of the smartphone [21,22]. Also, the magnetometer sensor can easily be disturbed in the presence of metallic objects [21]. The new methodology introduced in this paper intends to overcome these issues. 

## 3. System Implementation and Data Collection

**Figure 2:**
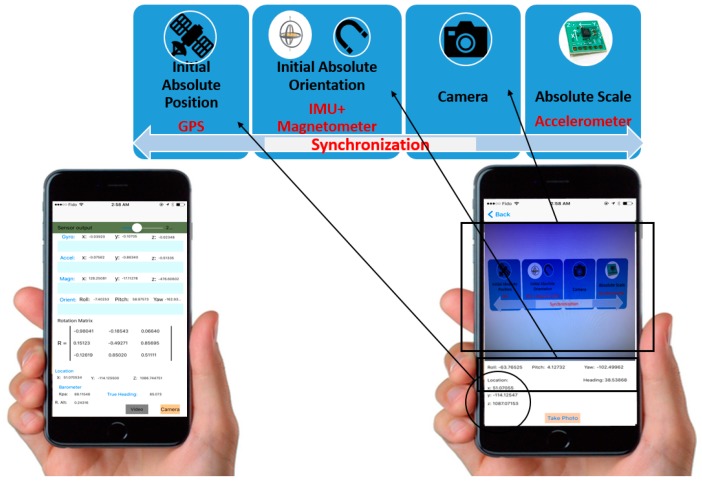
Developed iOS application.

An iOS software application was developed to capture and synchronize the images with their corresponding GPS and motion sensors measurements (location and orientation) at the time of exposure. Figure 2 shows a snapshot of the developed application, which was installed on an iPhone-6 equipped with a GPS receiver, 6-Axis IMU (3-axis gyroscope and 3-axis accelerometer), pedometer, compass, and barometer [23]. Furthermore, the iPhone-6 is already equipped with a high-resolution digital camera with a resolution of 8 megapixels.

## 4. Methodology

**Figure 3:**
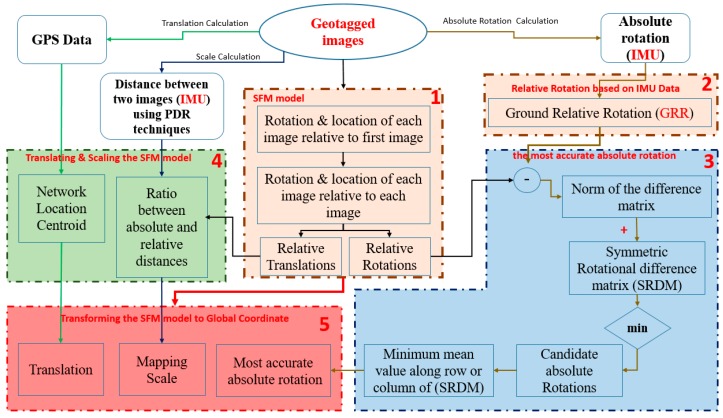
Methodology Workflow.

As illustrated in Figure 3, the methodologies begin by estimating the relative orientations (RO) w.r.t the first image using Structure from Motion (SfM), which provides an up-to-scale 3D model. Thus, the initial EOPs acquired by smartphone sensors are used to calculate global relative rotations (GRR) between each paired images. Then, each GRR is subtracted from the corresponding rotation acquired by the SfM; and the norm of each difference is used to build symmetric rotational difference matrix. The two corresponding relative rotations which have a close to zero difference are corresponding to the most accurate IMU measurements that associated with two images in the network. The accuracy of these two candidate absolute rotations is further examined where the most accurate one is then used to rotate the SfM model to the mapping coordinate frame while the absolute scale is determined using the ratio distance between two images, the relative distance is acquired by the SfM, and absolute distance obtained using pedestrian navigation techniques (e.g., steps detection).Also, the centroid of all the GPS locations associated with all images are calculated and used to translate the SfM model to the global coordinate. Once the initial EOPs are refined then these EOPs, along with geometric constraints, are entered into a free network bundle adjustment algorithm for reconstructing robust 3D objects. These steps are explained in details in the following subsections. 

### 4.1. Network Global Relative Rotation (GRR) Acquired by Smartphone Based on Motion Sensors

The main objective of this step is to find the relative rotation between each two images based on the absolute 3D rotations, which are directly acquired by smartphone’s motion sensors. These relative rotations are then used to evaluate the accuracy of each absolute rotation when compared with the SfM rotations.

**Figure 4:**
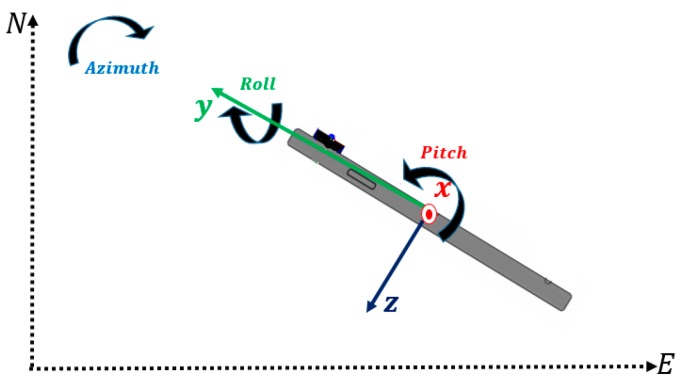
iPhone 6 Rotation Angles Definition.

The IMU along with magnetometers are used to obtain the direct 3D rotation of the smartphone instantly when the smartphone’s camera captures an image. The accelerometers sensor in the IMU are used to obtain the pitch and roll angles, which are rotation angles around the iPhone x-axis and y-axis respectively. Whereas, the magnetometer is used to derive the heading angle of the iPhone, which is measured w.r.t the iPhone y-axis as illustrated in Figure 4. 

These Euler angles are used to compute the rotation matrix $R_{b}^{m}$, which rotate the motion sensors measurements from the IMU (body) frame to the Global frame as expressed by Equation (1):

(1) $$R_{b}^{m}=R_{z}\left(Azimuth-90\right)\;\times \;R_{x}\left(Pitch\right)\;\times \;R_{y}\left(Roll\right)$$

In photogrammetry, the desired orientation is related to the involved camera frame. Therefore, the Euler angles derived by the IMU (inside the phone) are used to determine the photogrammetric orientation angles (e.g., omega, phi, and kappa) utilizing a boresight matrix as shown in Equation (2), where omega, phi, and kappa are the rotation angles around the camera x-axis, y-axis, and z-axis respectively:

(2) $$\mathrm{boresight}=\left(\begin{array}{ccc} 0 & -1 & 0\\ 1 & 0 & 0\\ 0 & 0 & 1 \end{array}\right)$$

These final absolute rotation angles are used to establish the Global Relative Rotation (GRR) between each two images in the network as expressed by Equation (3). For instance, if we have a network consisting of three images and we have the absolute EOPs acquired by the smartphone motion sensor, the RO between image 1 and image 2 can be calculated using these absolute EOPs:

(3) $$R_{j}^{i}={\left(R_{i}^{m}\right)}^{T}\;\times \;R_{j}^{m}\;\;R_{2}^{1}={\left(R_{1}^{m}\right)}^{T}\;\times \;R_{2}^{m}$$

where $R_{i}^{m}$ is the rotation between image (*i*) and mapping frame (Global frame). $R_{j}^{m}$ is the rotation between image (*j*) and mapping frame. $R_{j}^{i}$ is the GRR between image (*i*) and image (*j*). 

This procedure is repeated for each paired image in the network. These relative rotations then are compared to the relative rotations acquired by SfM algorithm in order to find the most accurate absolute IMU rotation, which finally will be used to rotate the SfM model to the mapping frame.

### 4.2. Network RO Recovery Using SfM

As mentioned earlier, closed form solutions (e.g., SfM) are much faster and do not require initial approximations of the unknowns compared to the traditional coplanarity conditions. Therefore, SfM is adopted for initial relative EOP estimation. The process of the SfM algorithm begins with the automatic computing of modified coplanarity condition using overlapped image pairs with at least eight or five matched tie points depending on the previous knowledge of the IOPs. The modified coplanarity condition is based on computing the essential matrix, which used to estimate the transformation parameters between each image pair. The initial EOPs of the network w.r.t the first image, are established via successive resections to compute the position and orientation for each image in the network, whereas, the initial 3D coordinates of the tie points are computed using successive intersection. These relative preliminarily EOPs and 3D coordinates of tie points are then refined using Bundle adjustments with adoptive (non-strict) outlier rejection tolerances [3,7,24]. 

**Figure 5:**
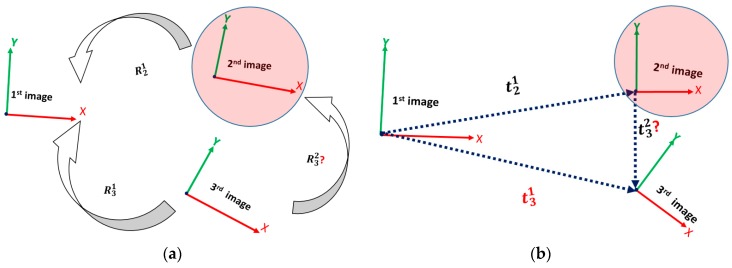
(**a**) Modified SfM Rotation, (**b**) Modified SfM Translation.

The main idea of this process is to compare the GRR derived from IMU with the corresponding RO obtained by SfM model. However, the GRR derived from IMU are computed for each paired image in the network. For example, the third image is considered a reference for the relative coordinate system based on IMU rotations (GRR), hence the GRR of any image is computed w.r.t the third image. However, the RO obtained by SfM are obtained with respect to the first image in the network. Therefore, new workflow is introduced to make each image in the network reference, one at a time, for the SfM model, while the EOPs of other images are computed relatively to the new reference image ith instead of 1st image. Therefore, Figure 5, Equations (4) and (5) illustrate the case where the second image is chosen to be the new reference instead of first image for the SfM model. 

The workflow begins by rotating each image to the chosen reference image, which is illustrated in Figure 5a and expressed by Equation (4):

(4) $$R_{i}^{x}={\left(R_{x}^{1}\right)}^{T}\;\times \;R_{i}^{1}\;\;R_{i}^{2}={\left(R_{2}^{1}\right)}^{T}\;\times \;R_{i}^{1}$$

where (1) is the first image that is originally used as reference for the network relative EOPs estimation using SfM. $R_{i}^{x}$ is the relative rotation between the new reference image and other images. $R_{x}^{1}$ is the relative rotation between the chosen new reference image (e.g., 2nd image in Figure 5a) and the first image. $R_{i}^{1}$ is the relative rotation between each image in the network and image (1). Then, the translation is redefined between each image and the reference one, as illustrated in Figure 5b and expressed by Equation (5):

(5) $$\begin{array}{l} \vec{t_{3}^{1}}=\vec{t_{2}^{1}}+\vec{t_{3}^{2}}\;\\ \vec{t_{3}^{2}}={\left(R_{2}^{1}\right)}^{T}\;\times \;\left(\vec{t_{3}^{1}}-\vec{t_{2}^{1}}\right)\;\\ \vec{t_{i}^{x}}={\left(R_{x}^{1}\right)}^{T}\;\times \;\left(\vec{t_{i}^{1}}-\vec{t_{x}^{1}}\right) \end{array}$$

where, $\vec{t_{3}^{1}}$ is translation vector between 3rd image and 1st image. $\vec{t_{2}^{1}}$ is translation vector between 2nd image and 1st image. $\vec{t_{3}^{2}}$ is translation vector between 3rd image and 2nd image. $R_{2}^{1}$ is the relative rotation between 2nd image and 1st image.

### 4.3. Comparing Different RO Matrices

**Figure 6:**
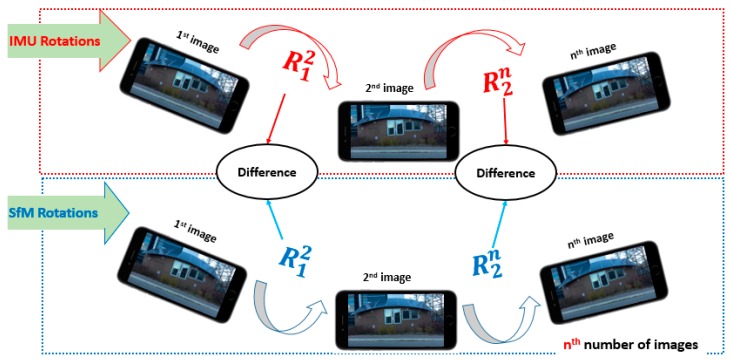
The difference between each corresponding relative rotation.

As mentioned earlier, the objective of this step is to identify the image that is directly geo-referenced with the most accurate IMU rotation angles, where it can be used to rotate the SfM model to the global frame. Therefore, once the GRR and the SfM’s relative rotation for each paired image in the network are obtained, the difference between each corresponding rotation is calculated using Equation (6) as illustrated in Figure 6. To represent the difference result with one value instead of the (3 by 3) matrix, the norm of the difference matrix is calculated using Equation (7). This value then is used to reconstruct a n by n matrix, where n is the number of images in the network:

(6) $$Dif_{i}^{j}=GRR_{i}^{j}-R_{i}^{j}$$

where Dif  is the difference between each corresponding relative rotation matrices in the network. $GRR_{i}^{j}$ is the relative rotation acquired based on the smartphone motion sensor measurements. $R_{i}^{j}$ is the relative rotation obtained using SfM algorithm.

The difference (Dif) matrix is computed for each two images in the network. Each Dif matrix is represented as one value using matrix norm as expressed by Equation (7):

(7) $$N_{i}^{j}=norm\left(Dif_{i}^{j}\right)$$

#### Symmetric Rotational Differences Matrices

The main objective of this step is to determine the most accurate absolute rotation among the candidate IMU rotations in order to use it to rotate the SfM model to the global frame. Therefore, based on Equations (6) and (7), the norm value is used to reconstruct a (n by n) matrix that consists of the difference, where n is the number of images in the network. For example, for a network consisting of three images, the norm of the difference matrix ($N_{i}^{j}$) between each two corresponding relative rotations is calculated using Equations (6) and (7) and placed in the cell that is located between these images as shown in Table 2. Based on the off-diagonal elements in Table 2, the two corresponding relative rotations with difference value close to or equal to zero are selected as the candidate for rotating the SfM model. 

However, one of the candidate rotation needs to be chosen to rotate the SfM model. In the case of considering image 3 and image 2 to be associated with the most accurate IMU rotation (with off diagonal value of 0.005 in the example in Table 2). Equation (8) is used to determine the most accurate IMU rotation by calculating the mean of rows correspond to image 3, and the mean of the column corresponds to image 2 in matrix DD. As a result, the image associated with less mean error value corresponds the most accurate absolute IMU rotation in the network. This absolute IMU rotation is then used to rotate the SfM model:

(8) $$\begin{array}{l} \theta_{row}=mean\left(DD\left(:,image2\right)\right)\;\\ \theta_{column\;}=mean\left(DD\left(image3,:\right)\right) \end{array}$$

where, $\theta_{row}$ is the mean of the error value along the column that is corresponding to image 2. $\theta_{column\;}$ is the mean of the error value along the row that is corresponding to image 3.

### 4.4. Transforming the SfM Relative Model to the Mapping Coordinate

The transformation of a model from one coordinate to another requires the prior knowledge of seven parameters (i.e., three translations, three rotations, and a uniform scale) [2,25]. Therefore, the process of transforming the SfM model to the global coordinates is organized in the following order: SfM rotation, scale determination, and centroid of GPS solutions calculation.

#### 4.4.1. SfM Rotation to Global Coordinate

As shown in Section 4.3, the most accurate absolute rotation derived by the smartphone IMU and magnetometer is determined and used to rotate the SfM model, which is established w.r.t the image that corresponds to the most accurate absolute rotation. 

#### 4.4.2. Centroid of GPS Solutions

The GPS chipset built into most smartphones does not provide raw measurements, such as pseudorange or carrier phase. The user can only log the final position solution with its standard deviation as calculated by the iOS or Android API system [20]. As a result, the user is limited by this solution, whereby no further improvements can be made. Therefore, in this method, each GPS solution that is used to geotag a captured image is used to calculate the network centroid based on the weighted average. The weight is derived based on the provided standard deviation of each solution. 

#### 4.4.3. Mapping Scale Acquisition from Smartphone

The mapping scale can be determined using the ratio between two distances. One of which is obtained in the SfM arbitrary coordinate and the other one is calculated in the actual mapping (global) coordinate as expressed by Equation (9):

(9) $$\mathrm{Scale}=\frac{D_{\mathrm{SfM}}}{D_{\mathrm{mapping}}}$$

These distances can be calculated either between two points or two camera locations. Hence, the distance between two images is employed in this method. The mapping scale can be acquired using a traditional technique, which measures the distance between two points using measuring tape or imaging an object with a known length. The two distances are used to derive the scale value as shown in Equation (9). Although this technique is the most accurate one, it requires user interference in the process. Therefore, some pedestrian navigation techniques (e.g., step detection and step length estimation) are adapted to calculate the scale automatically. Lee et al. developed a robust step detection algorithm against the dynamics of smartphones, which is based on the 3D magnitude of accelerometer measurements. This algorithm begins by filtering the acquired data using a low-pass filter, and then extracting the measurements corresponding to the smartphone’s motion. Then, the extracted motions epochs are classified as peak-valley relationships using adaptive thresholds that are based on step average and step standard deviation. Furthermore, the adaptive time threshold is used to correct the candidate peak and valley. This algorithm shows 98.6% step detection accuracy [26]. 

**Figure 7:**
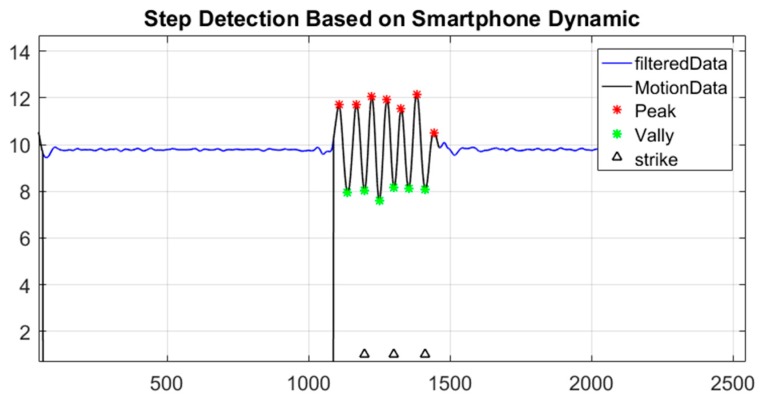
The Step Detection Result.

As a result, this algorithm is adopted in this method to detect the user steps between two successive captured images as illustrated in Figure 7, where the user is constrained to walk in a straight line and the strike consists of two steps. Hence, the detected peaks and valleys are sorted in ascending order, and the acceleration between the first and last step is double integrated to calculate the distance between two images. This distance is used to represent the denominator in Equation (8).

The main challenge of validating the proposed scale method over a known reference distance is that it is impossible to start and end exactly over the start and end points of the reference distance. Therefore, image-based technique is introduced as well to determine the possible shift between the reference distance and the start and end distance estimated using the accelerometers. 

**Figure 8:**
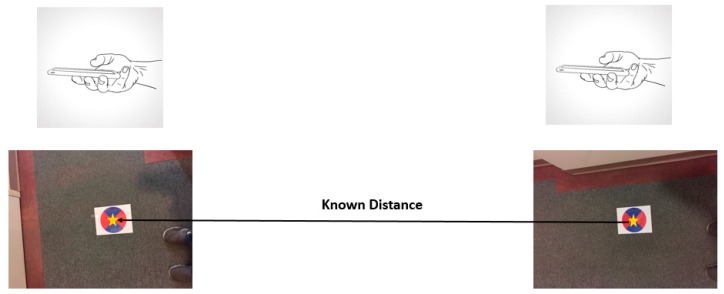
The Step Detection Result.

Two color coded targets are placed on ground with a 7.22 m distance between them. The diameter ($D_{T}$) of each target is at 20 cm. At each validation experiment, the user is asked to capture two images, one for the first target and another for the second target, as shown in Figure 8. Then, a morphological image classification technique is used to extract the centroid and the diameter ($D_{i}$) of each target, whereby the scale ($\lambda_{i}$) is determined by the ratio between the actual target diameter and the image diameter of the same target. The shift between the centroid of each target and the camera perspective center position at the time of exposure then are calculated using Equation (10):

(10) $$\begin{array}{l} \lambda_{i}=\left(\frac{D_{T}}{D_{i}}\right)\;\\ s_{1}\;=\left(c_{1}-\left(\frac{w}{2}\right)\right)\;\times \;\lambda_{1}\;\\ s_{2}\;=\left(c_{2}-\left(\frac{w}{2}\right)\;\right)\;\times \;\lambda_{2}\; \end{array}$$

where, $s_{1}$ and $s_{2}$ are the actual horizontal shifts from the smartphone camera perspective center to the center of the target fixed on the ground for targets one and two respectively, $c_{1}$ and $c_{2}$ are the image space coordinates for the centroids of targets one and two, respectively, measured in pixels. w is the image format width, measured in pixels. 

The two shifts are then subtracted from the known distance to determine the exact true distance for each validation test as expressed by Equation (11):

(11) $$True\;Distance\;\left(i\right)=\frac{\left(7.22+s_{1}-s_{2}\right)}{100}\;$$

Several validation tests were conducted to assess the performance of the distances measured by the accelerometers and the result indicated the presence of approximately 7 cm errors between the known truth distance and the proposed method.

### 4.5. Geometric Constraints in Bundle Adjustment

**Figure 9:**
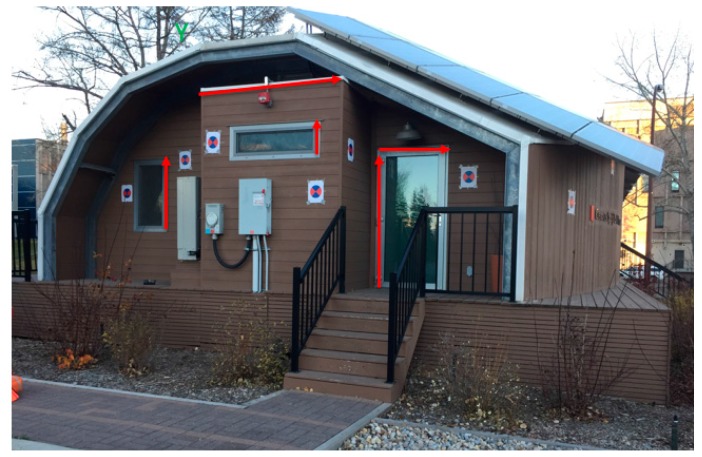
Straight Linear Feature Constraints.

As mentioned previously, geometric information (i.e., vertical, and horizontal linear features) that are visible in the captured images can be used as constraints to the bundle adjustment. These constraints are independently determined observations, capable of being added to the system equations in the normal matrix to ensure more reliable and higher quality solutions [18]. Therefore, vertical and horizontal line constraints are used to enhance the final bundle adjustment result as expressed using Equations (12) and (13) [14]. These constraints are measured in the object space domain and are added to the object space unknown parameters. As illustrated in Figure 9, the only change between any two points located along a vertical line is the height as expressed by Equation (11). The East and North dimensions for these points are similar as expressed by Equation (12). Likewise, any two points along the horizontal line have the same value for height, while the East and North dimensions for each point are different as expressed by Equation (13):

(12) $$X_{i}-X_{j}=Y_{i}-Y_{j}=0$$

(13) $$Z_{i}-Z_{j}=0$$

where i,j are any two points on a straight line.

### 4.6. Free Network Adjustment with Geometric Constraint

The 3D object reconstruction is conducted using collinearity equations, which defines the mathematical relationship between the image and the ground coordinate system [2,25] as expressed by Equations (14a) and (14b):

(14a) $$x_{a}=x_{p}-c\frac{r_{11}\left(X_{A}-X_{0}\right)+r_{12}\left(Y_{A}-Y_{0}\right)+r_{13}\left(Z_{A}-Z_{0}\right)}{r_{31}\left(X_{A}-X_{0}\right)+r_{32}\left(Y_{A}-Y_{0}\right)+r_{33}\left(Z_{A}-Z_{0}\right)}+∆x$$

(14b) $$y_{a}=y_{p}-c\frac{r_{21}\left(X_{A}-X_{0}\right)+r_{22}\left(Y_{A}-Y_{0}\right)+r_{23}\left(Z_{A}-Z_{0}\right)}{r_{31}\left(X_{A}-X_{0}\right)+r_{32}\left(Y_{A}-Y_{0}\right)+r_{33}\left(Z_{A}-Z_{0}\right)}+∆y$$

where (${\;r}_{11}$ to $r_{33}$) represents the element of the rotation matrix, c is the perspective distance of the camera. The image coordinates of the principal point are $x_{p}$, $y_{p}$; the image coordinates of the object point are x_a_, y_a_; the perspective centre ground coordinates are $X_{0}$, $Y_{0}$ and $Z_{0}$, and the object point ground coordinates are $X_{A}$, $Y_{A}$ and $Z_{A}$.

The study in this paper aimed to develop a stable low-cost direct geo-referencing system that can be operated in any outdoor environment without the need for GCPs whereas, the bundle adjustment requires a fixed reference consisting of seven parameters, which defines the network datum. The datum is traditionally defined by at least three GCPs for indirect geo-referencing or very accurate GPS/IMU systems for direct geo-referencing. To overcome the datum deficiency issue, a free bundle-adjustment procedure was used. This procedure uses the inner constraint matrix (G) to remove the rank defect of the normal matrix [27,28,29]. This constraint fits the network onto the estimated initial ground coordinate of the tie point values as shown in Equation (15). Hence, the inner constraint matrix accounts for the datum seven parameters (three translations, three rotations, and scale):

(15) $$G=\left(\begin{array}{ccccccc} 1 & 0 & 0 & 0 & -Z_{A} & Y_{A} & X_{A}\\ 0 & 1 & 0 & Z_{A} & 0 & -X_{A} & Y_{A}\\ 0 & 0 & 1 & -Y_{A} & X_{A} & 0 & Z_{A} \end{array}\right)$$

where $X_{A},Y_{A}$ and $Z_{A}$ are the initial values for the ground coordinate of the tie point, the free bundle adjustment is a nonlinear least square technique used to calculate the EOPs, the desired 3D object point coordinate, and the IOPs utilizing the collinearity condition and the inner constraint as expressed by Equation (16):

(16) $$\left(\begin{array}{c} \delta {\hat{x}}_{EOPs}\\ \delta {\hat{x}}_{OPs}\\ K \end{array}\right)={\left(\begin{array}{ccc} A^{T}{}_{EOPs}R^{-1}A_{EOPs}+R_{Eops} & A^{T}{}_{EOPs}R^{-1}A_{OPs} & 0\\ A_{OPs}{}^{T}\;R^{-1}\;A_{EOPs} & A^{T}{}_{OP}R^{-1}A_{OPs}+R_{Ops}+A_{C}^{T}\;R^{-1}A_{c} & G\\ Sym. & & 0 \end{array}\right)}^{-1}*\left(\begin{array}{c} A^{T}{}_{EOPs}\;R^{-1}\;w+R_{Eops}\;+w_{Eops}\\ A^{T}{}_{OPs}R^{-1}w+R_{Ops\;}w_{Ops}+A_{C}^{T}\;R^{-1}w_{c}\\ 0 \end{array}\right)$$

where, $\delta \hat{x}$ is the unknown parameters vector, A is the design matrix, R is the weight matrix, w is the misclosure vector. The linear features constraints are denoted subscript (c), and the subscript EOPs represents the EOPs parameters. Also, the subscript OPs represents the object’s point parameters. K is the Lagrange multiplier. 

## 5. Experimental Results

**Figure 10:**
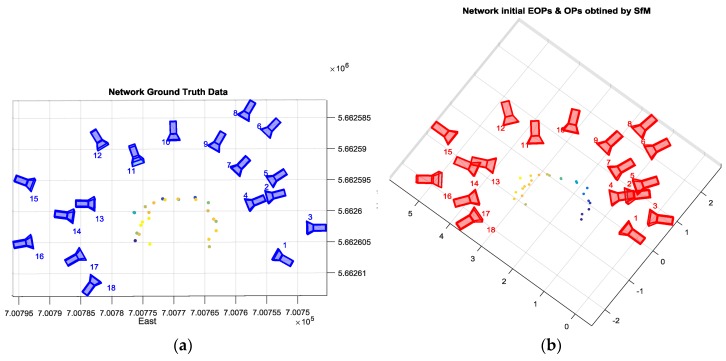
(**a**) The reference 3D model; (**b**) 3D model obtained by SfM.

To test the developed methodologies, an iPhone 6 was used to collect close range images along with their corresponding motion sensors measurements. As shown in Figure 10a, the collected images emulate a network consisting of 18 images and 25 OPs. To test the accuracy of the proposed methodologies, the reference EOPs of the images and the OPs were measured using Total Station, whereby the position of each camera station was determined within 1 cm and the rotation angles were within 30 arc second accuracies based on the Total Station’s measurement accuracy. Furthermore, the 3D tie points’ position accuracy was within 1 cm. 

The experimental data were collected over a small building located in a harsh environment, for both the GPS and magnetometers, where the building is surrounded by high buildings which could introduce multipath in the GPS signals. Moreover, the building is located close to large parking, whereby, the presence of vehicles causes magnetometers measurements. Such environment is very challenging for all MMS especially those with low-cost sensors.

According to Clive [30], network design is the technique to ensure the reliability and precision of the bundle adjustment, especially the free network adjustment approach. The network design can be classified to four orders as stated by Grafarend [31] and Clive [30]:

- Zero-Order Design (ZOD), which is associated with datum problem.
- First-Order Design (FOD), which is associated with optimum network configuration.
- Second-Order Design (SOD), which is associated with optimum number of observations and their corresponding weighting scheme.
- Third-Order Design (TOD), which is associated with enhancing the network by adding more images, observations, and object points.

These precautions were considered in the planning stage of this experiment, more specifically the ZOD, FOD, and SOD, which are discussed in Section 5.3.

### 5.1. Initial EOPs and OPs 3D Coordinate Correction

The initial EOPs and OPs were determined using the SfM algorithm, which provided a good shape for the network compared to the ground data collected using Total Station as shown in Figure 10, but these initial parameters were in an arbitrary frame of coordinates. Therefore, this result needed to be scaled, rotated, and shifted to the global coordinate as discussed in the proposed method. 

**Figure 11:**
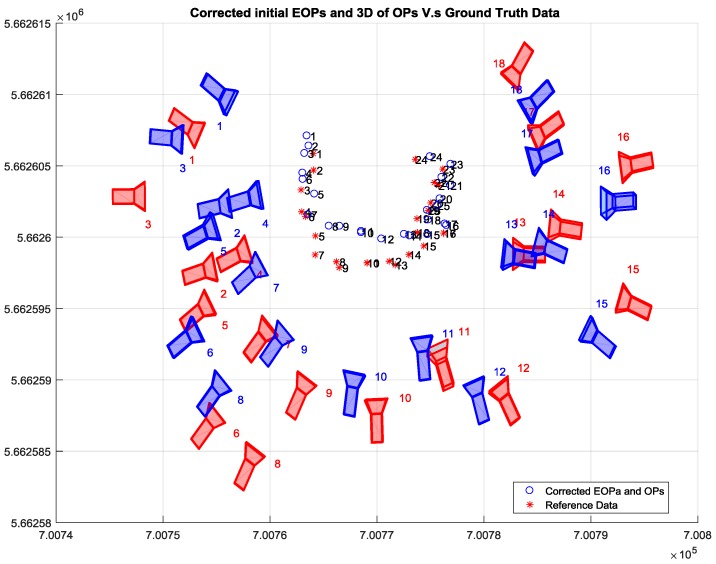
The final corrected initial EOPs and Ops.

First the scale was determined using the distance between two successive images; then, the SfM model was rotated using the most accurate IMU data corresponding to one of the images. Finally, the GPS centroid was utilized to translate the network. The results of the transformation process and the corrected initial EOPs are illustrated in Figure 11. 

**Figure 12:**
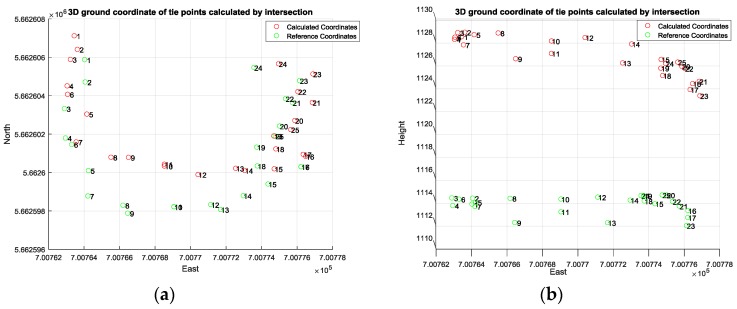
(**a**) Horizontal coordinate of OPs based on intersection; (**b**) Vertical coordinate of OPs based on intersection.

Then, the well-known intersection method was conducted to calculate the 3D coordinates of the desired tie points utilizing the image measurements and the corrected initial EOPs of the involved camera stations as illustrated in Figure 12a,b.

Table 3 shows the accuracy of the corrected initial EOPs of the network using the proposed relative orientation method compared to the ground truth data acquired using Total Station. Although the proposed method attempted to overcome the GPS random errors by employing the GPS solutions centroid, the existing error was within the accuracy of the single point positioning (SPP) solution provided by iOS system. In addition to the horizontal errors that occurred due to the poor accuracy of the low-cost GPS and the uncertainty of scale calculation, the SPP method provided inaccurate height estimation compared to its horizontal accuracy. Table 4 lists the absolute difference between the initial Object Points (OPs) and the absolute reference data acquired by Total station.

### 5.2. Free Network Adjustment with Geometric Constraint Cooperation

**Figure 13:**
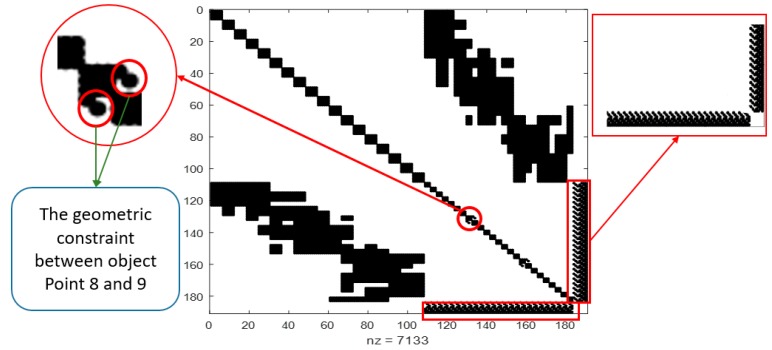
Normal matrix with all object points constrained by inner matrix G and Geometric Constraint.

Although, the initial EOPs were corrected using the proposed relative orientation, they still contained some errors due to the uncertainty of the scale automatic determination and the low-cost sensor errors. Therefore, the corrected initial EOPs and OPs became the input to the free network adjustment; and all the selected OPs were included in the inner constraints matrix to fix the network as illustrated in Figure 13. Moreover, three vertical and three horizontal linear features were visible in most of the captured images and distributed in different locations around the object, which were chosen to improve the final solution as illustrated in Figure 13. 

**Figure 14:**
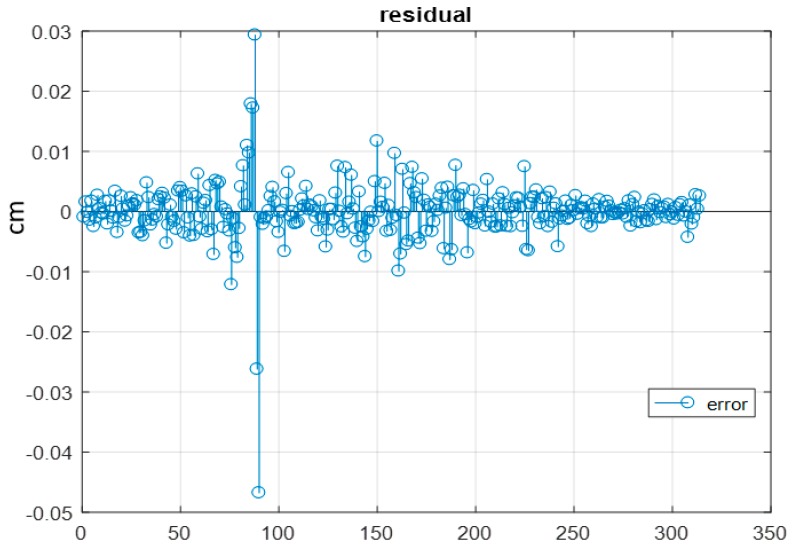
Residual at the final iteration.

Each of these lines is defined by two points that were measured manually to ensure the accuracy of extracting the same line in the overlapped images. The solution was obtained using the free network adjustment two times; one with and the other without adding the geometric constraints equations to the normal matrix. The inclusion of the linear constraints provided more accurate results as illustrated in Table 5. Furthermore, the final iteration residual was very low, which yield to good convergence of the solution as shown in Figure 14.

Table 5 lists the absolute difference between the calculated final OPs and the absolute reference data. The incorporation of the linear features constraints into the free network adjustment improved the absolute position in East, North, and the height by 8, 9 and 2 cm, respectively. This improvement is associated with the absolute position of the OPs. This result is expected since free network adjustment enhances the relative accuracy more than the absolute position. 

**Figure 15:**
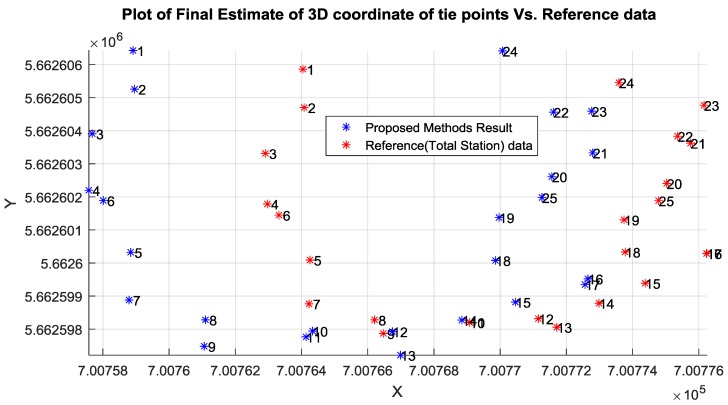
Estimated horizontal coordinate of tie point.

**Figure 16:**
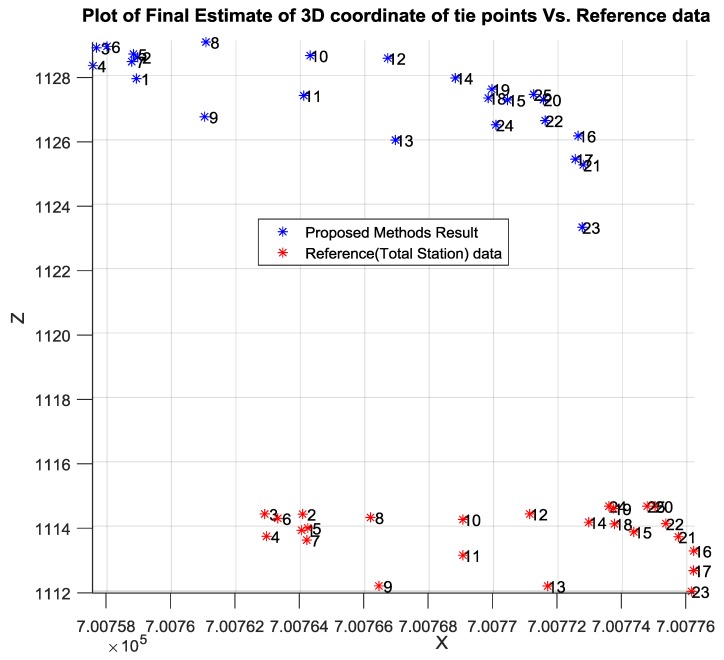
Estimated vertical coordinates of tie point.

As can be observed from Table 5 and Figure 15 and Figure 16, the calculated east and north ground coordinates of the tie points were even better than the GPS accuracy when used directly without any enhancement. However, the vertical accuracy was worse than the horizontal accuracy as illustrated in Figure 15 and Figure 16, which is common normal case when acquiring the GPS solution based on the praise point positioning technique. Overall, the proposed method mitigated the GPS random error by relying on the centroid of all the solutions, which were calculated based on the weighted average rather than including all the GPS solutions. 

### 5.3. Impact of Network Design on the Proposed Method

**Figure 17:**
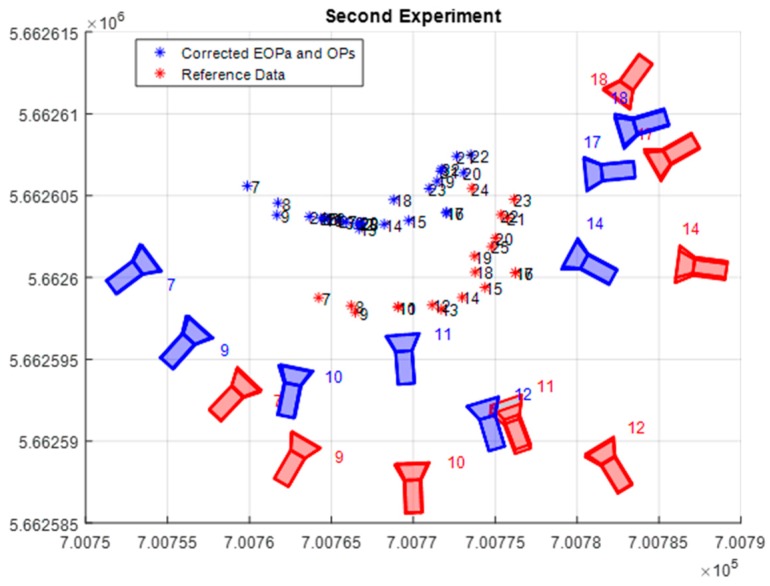
Second experiment final corrected initial EOPs and Ops.

To verify the robustness of the proposed methods, especially the relative accuracy; eight images out of the previously used network are considered as a new network as illustrated in Figure 17. Furthermore, the images that were found to correspond to the most accurate absolute rotations in the previous experiment are removed from the new network. Also, a new set of nine object points are added to the new network. Since the proposed methodologies involved several types of observations with varied levels of uncertainty, the impact of changing any of these observations uncertainty on the final solution was considered. Therefore, the new experiment was divided into two scenarios, namely free network adjustment with and without linear features constraints. 

#### 5.3.1. Free Network Adjustment without Linear Feature Constraints

In this section, the ZOD is considered to determine the network datum. Also, the impact of the mean precision (RMS) of the final 3D OPs by changing the standard deviation (σ) of the photo/image measurements and the number of OPs was studied as shown in Table 6. 

Based on Table 6, there is a direct linear relationship between the image observations standard division (σ)  and the output 3D OPs coordinate mean precision (RMS). Approximately, the mean precision of the OPs coordinates degrades linearly when the image observations standard division (σ) increases. Also, it can be seen that, as the number of OPs decreases, the mean precision of 3D OPs final solution increases. 

#### 5.3.2. Free Network Adjustment with Linear Features Constraints

In this section, the effect of changing the number of constraints on the solution is analyzed while the image observation standard deviation remains unchanged from the previous scenario. Also, the constraint observations weight was set to be smaller by five orders of magnitude compared to the image observations, similar to the approach in Gerke [12]. Table 7 shows the RMS of the 3D OPs final solution result when changing the number of linear feature constraints. The incorporation of linear features constraints enhanced the mean precision of the 3D OPs, while increasing the number of constraints further enhanced the RMS. 

The results obtained from the proposed methodology is, however, limited to the network design used in this research. The network configuration influences the OPs precision, which thereby affects the relative accuracy of the introduced methodology. However, its absolute accuracy depends on the quality of low-cost GPS, which can vary between one to five meters in ideal conditions.

## 6. Conclusions

This paper introduced a robust smartphone-based MMS in conjunction with a new proposed workflow that overcomes the drawbacks of random errors associated with the smartphone motion sensors and poor GPS accuracy. The proposed workflow corrects the initial EOPs based on the relative orientation of the captured images using the SfM algorithm. These relative orientations then are used to validate the absolute orientations of these images, which are obtained directly by the smartphone’s motion sensors measurements (i.e., accelerometers and magnetometers). First, the relative rotations are calculated w.r.t the first image in the network utilizing the SfM algorithm. Then, this procedure is repeated to make each image in the network a reference for the SfM model, thereby achieving the relative rotation between each two images in the network. However, to evaluate the absolute rotation for each image that are acquired directly by smartphones, these rotations are used to derive the relative rotation between each two images in the IMU domain. Hence, the difference between each corresponding relative rotation obtained in different domains are evaluated to identify the images that have the most accurate relative rotation. Then, based on statistical evaluation, the absolute rotation of one of these images is used to rotate the SfM model established w.r.t this image. Subsequently, this model is scaled using the ratio between two corresponding distances, where one distance is acquired based on a step detection algorithm utilizing accelerometer measurements along with a pedestrian navigation technique, and the other distance is measured from the selected SfM model. The network global absolute centroid is calculated using the acquired GPS solutions for all the captured images, and this centroid is used to translate the centroid of the SFM model to the global coordinate. 

Finally, the corrected initial EOPs are incorporated with the linear features constraints, and refined using free network bundle adjustment. The proposed methodology was applied to a small building that has well distributed GCPs all around the building facade. The proposed system and methodology showed promising results for applications that require three to five meters absolute accuracy. Although this system provided reasonable accuracy compared to the employed sensors and their uncertainty, future research should include finding a way to extract the scale without the user step length measurement, which can introduce large error in case the user step length is changed while walking between two images. 

## Figures and Tables

**Table 1:** Example of iPhone 6 chipsets accuracy over known distance.

Trajectory	Test 1	Test 2
reference distance	69.789 m	69.789 m
iPhone 6 GPS distance	73.9 m	199 m

**Table 2:** The difference between each corresponding relative rotation result.

DD	Image 1	Image 2	Image 3
Image 1	……‥	$N_{1}^{2}$	$N_{1}^{3}$
Image 2	$N_{2}^{1}$	……‥	$N_{2}^{3}$
Image 3	$N_{3}^{1}$	0.005	……‥

**Table 3:** The corrected initial position of the Station’s camera absolute accuracy.

Error	East (m)	North (m)	Height (m)	Error	East (m)	North (m)	Height (m)
Image 1	−3.01	−2.39	−14.66	Image 10	2.43	−1.88	−12.86
Image 2	−1.41	−4.59	−14.62	Image 11	1.97	−0.57	−12.03
Image 3	−3.50	−4.15	−15.18	Image 12	2.71	−0.05	−11.51
Image 4	−0.99	−4.09	−14.30	Image 13	0.91	0.30	−10.38
Image 5	−0.36	−5.65	−14.84	Image 14	1.43	1.55	−9.70
Image 6	2.13	−6.26	−15.09	Image 15	3.36	2.54	−8.67
Image 7	1.03	−4.69	−14.24	Image 16	1.63	2.72	−8.16
Image 8	3.37	−5.12	−14.66	Image 17	0.17	1.85	−9.49
Image 9	2.32	−3.66	−13.84	Image 18	−1.84	2.52	−9.42
RMS							
East (m)			North (m)			Height (m)	
2.16			3.52			12.66	

**Table 4:** The determined absolute accuracy of the initial Object Points (OPs).

Error	East (m)	North (m)	Height (m)	Error	East (m)	North (m)	Height (m)
OP 1	0.58	−1.25	−14.64	OP 13	−0.85	−2.15	−13.97
OP 2	0.44	−1.70	−14.52	OP 14	−0.08	−1.32	−13.66
OP 3	−0.34	−2.56	−14.46	OP 15	−0.35	−0.79	−12.61
OP 4	−0.09	−2.72	−14.56	OP 16	−0.27	−0.54	−11.10
OP 5	0.09	−2.94	−14.68	OP 17	−0.13	−0.64	−11.18
OP 6	0.23	−2.61	−14.18	OP 18	−1.04	−0.88	−10.97
OP 7	0.66	−2.81	−14.14	OP 19	−0.96	−0.58	−11.15
OP 8	0.68	−2.50	−14.47	OP 20	−0.86	−0.29	−11.25
OP 9	−0.05	−2.91	−14.34	OP 21	−1.12	−0.01	−10.90
OP 10	0.54	−2.12	−13.83	OP 22	−0.70	−0.36	−11.63
OP 11	0.54	−2.21	−13.85	OP 23	−0.75	−0.35	−11.35
OP 12	0.72	−1.57	−13.96	OP 24	−1.38	−0.19	−11.55
				OP 25	−0.87	−0.34	−11.61
RMS							
East (m)			North (m)			Height (m)	
0.68			1.76			13.06	

**Table 5:** The calculated final 3D ground coordinates of the accuracy of the tie points.

Error	East (m)	North (M)	Height (m)	Error	East (m)	North (M)	Height (m)
OP 1	−0.71	−2.74	−14.16	OP 13	0.64	−0.86	−13.05
OP 2	−0.63	−2.67	−14.26	OP 14	−0.05	−0.83	−12.79
OP 3	−0.36	−2.77	−14.58	OP 15	−0.16	−0.61	−12.36
OP 4	0.05	−2.65	−14.65	OP 16	−0.30	−0.22	−11.79
OP 5	0.24	−2.39	−14.54	OP 17	−0.16	−0.11	−11.73
OP 6	−0.02	−2.61	−14.63	OP 18	−0.36	−0.85	−12.27
OP 7	0.52	−2.33	−14.63	OP 19	−0.63	−1.04	−12.11
OP 8	0.35	−2.01	−14.29	OP 20	−0.87	−0.95	−11.68
OP 9	0.89	−1.81	−14.19	OP 21	−1.19	−0.40	−10.72
OP 10	0.24	−1.49	−13.69	OP 22	−0.71	−1.33	−11.72
OP 11	0.53	−1.41	−13.64	OP 23	−0.73	−0.52	−10.71
OP 12	0.07	−1.14	−13.26	OP 24	−1.24	−1.55	−11.27
				OP 25	−0.79	−0.92	−11.82
RMS							
East (m)			North (m)			Height (m)	
0.60			1.67			13.04	

**Table 6:** The effect of changing observation weight on 3D OPs final solution RMS.

Number of Tie Points	Image Observation σ	Mean Precision of 3D OPs Final Solution (RMS)		
		X	Y	Z
26	1/2 pixel	0.004	0.004	0.002
	2 pixels	0.018	0.015	0.007
	5 pixels	0.044	0.037	0.018
17	1/2 pixel	0.007	0.005	0.002
	2 pixels	0.027	0.02	0.009
	5 pixels	0.068	0.05	0.024

**Table 7:** The effect of changing number of constraints on 3D OPs final solution RMS.

Number of Vertical Linear Features	Number of Horizontal Linear Features	Image Observation σ	Mean Precision of 3D OPs Final Solution		
			X	Y	Z
		1/2 pixel	0.004	0.004	0.002
1	1	2 pixels	0.018	0.015	0.007
		5 pixels	0.044	0.037	0.018
2	2	1/2 pixel	0.0033	0.0033	0.0014
		2 pixels	0.013	0.012	0.005
		5 pixels	0.033	0.033	0.014
4	3	1/2 pixel	0.002	0.002	0.001
		2 pixels	0.008	0.008	0.004
		5 pixels	0.021	0.022	0.010
